# Pingyangmycin-loaded drug-eluting beads transarterial embolisation for giant cavernous haemangioma of the liver: A case report

**DOI:** 10.1016/j.heliyon.2024.e36514

**Published:** 2024-08-17

**Authors:** Yonghua Bi, Jianzhuang Ren, Xinwei Han

**Affiliations:** Department of Interventional Radiology, The First Affiliated Hospital of Zhengzhou University, Zhengzhou, China

**Keywords:** Giant cavernous haemangioma, Computed tomography, Transarterial embolisation, Drug-eluting bead, Pingyangmycin

## Abstract

**Purpose:**

Cavernous haemangioma of the liver (CHL) is the most common venous malformation of the liver. Surgical resection is considered the gold standard for large symptomatic haemangiomas. Transarterial embolisation has demonstrated acceptable efficacy with lower rates of morbidity and mortality. We report the first case of a 59-year-old man with a giant CHL treated using pingyangmycin drug-eluting bead transarterial embolisation (DEB-TACE).

**Material and methods:**

A 59-year-old man presented to our hospital with cough and sputum, most probably related to the mass effect of the haemangioma and secondary lung collapse. Computed tomography (CT) revealed a 187.5 mm × 142.7 mm cavernous haemangioma located in the right lobe of the liver. He underwent DEB-TACE, and a 2.6-F microcatheter was used to selectively catheterise the right hepatic artery. One vial of 300–500-μm CalliSpheres microspheres loaded with 8-mg pingyangmycin and two vials of 100–300-μm microspheres were injected through the microcatheter until the disappearance of CHL staining.

**Results:**

The patient experienced mild abdominal pain on the second day after embolisation. A reduction in CHL size to 106.7 × 141.3 mm was observed on the 1.1-month follow-up CT. We performed a second similar DEB-TACE, which resulted in further size reduction to 83.1 × 50.1 mm, as detected on the follow-up CT at 4.6 months. At the 8.7-month follow-up, his clinical symptoms improved with no cough or sputum and the CHL size further reduced to 63.2 × 55.8 mm.

**Conclusion:**

We report the first case of a giant CHL treated using DEB-TACE. Although DEB-TACE may be an effective and safe alternative for treating of giant CHL, an *in vitro* study on the efficient loading and binding of pingyangmycin with microspheres and more comparative studies with larger samples are required to further confirm its safety and efficacy.

## 1 Introduction

Cavernous haemangioma of the liver (CHL) is the most common venous malformation of the liver. Surgical resection is considered as the gold standard for large symptomatic haemangiomas [1]. Transarterial embolisation has demonstrated acceptable efficacy with lower rates of post-procedural morbidity and mortality [2]. Drug-eluting bead (DEB) transarterial chemoembolisation (DEB-TACE) has gradually been used as locoregional therapy, and might have a higher complete response rate, disease control rate and tumor necrosis rate than conventional transarterial chemoembolisation (cTACE) for unresectable hepatocellular carcinoma [3,4]. Nevertheless, there still exists controversy regarding a higher complete response rate of DEB-TACE [5]. Herein, we report the first case of a 59-year-old man with a giant CHL treated using DEB-TACE to examine whether the necrosis and atrophy of the CHL were more dramatic.

## 2 Case presentation

The institutional review board approved this case report. Written informed consent was obtained from the patient for the publication of the case details and any accompanying images.

**Fig. 1:**
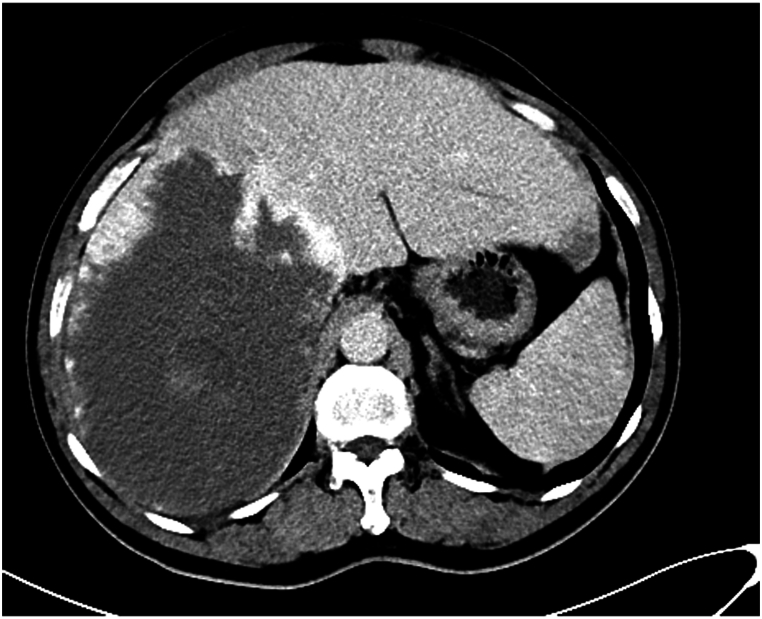
CT image obtained before DEB-TACE revealed a 187.5 × 142.7 mm CHL in the right lobe of the liver.

A 59-year-old man presented to our hospital with cough and sputum, most probably related to the mass effect of the hsemangioma and secondary lung collapse. Computed tomography (CT) revealed a 187.5 × 142.7 mm CHL located in the right lobe of the liver (Fig. 1). The patient felt anxious about the giant CHL and requested effective treatment; however, he refused surgical resection due to fear of considerable trauma and possible complications. Therefore, we performed DEB-TACE anticipating a better necrosis of the CHL. No medical, familial or psycho-social histories were available for this patient.

**Fig. 2:**
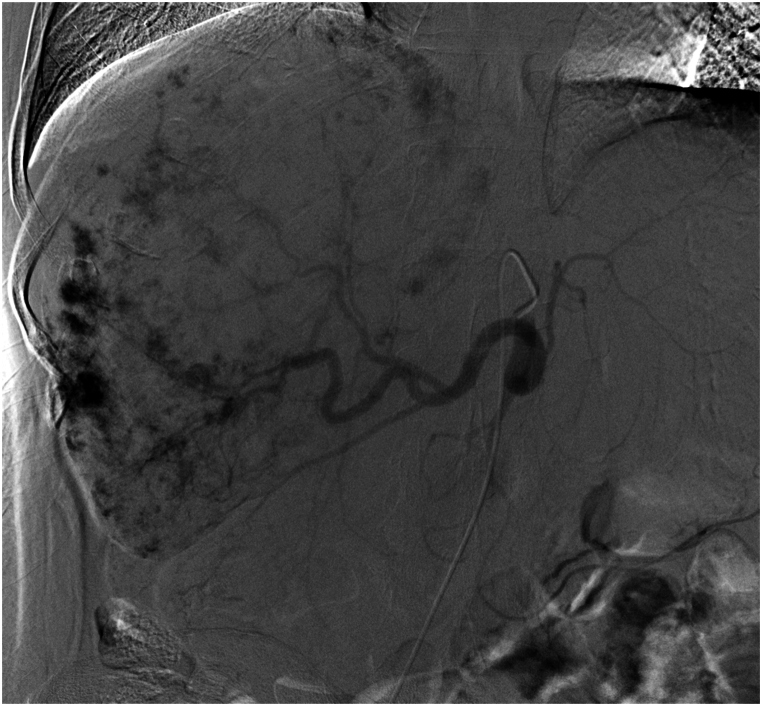
Angiographic image obtained during the first DEB-TACE revealed a giant CHL staining in the right lobe of the liver that was supplied by the right hepatic artery.

The right femoral artery was punctured, and a 5-F right hepatic catheter (Cook, Inc, Bloomington, Indiana) was inserted to examine the common hepatic artery. A giant CHL staining was detected in the right lobe of the liver that was supplied by the right hepatic artery (Fig. 2). A 2.6-F microcatheter (Suzhou Hengrui Callisyn Biomedical Co., Ltd., Suzhou, China) was used to selectively catheterise the right hepatic artery. Before embolisation, one vial of 300–500-μm CalliSpheres microspheres (Suzhou Hengrui Callisyn Biomedical Co., Ltd., Suzhou, China) was loaded with 8 mg of pingyangmycin for 5–10 min. However, due to the large size of the CHL, embolisation was not sufficient with one vial of DEBs; hence, two vials of 100–300-μm microspheres (Suzhou Hengrui Callisyn Biomedical Co., Ltd., Suzhou, China) were injected through the microcatheter until the disappearance of CHL staining.

**Fig. 3:**
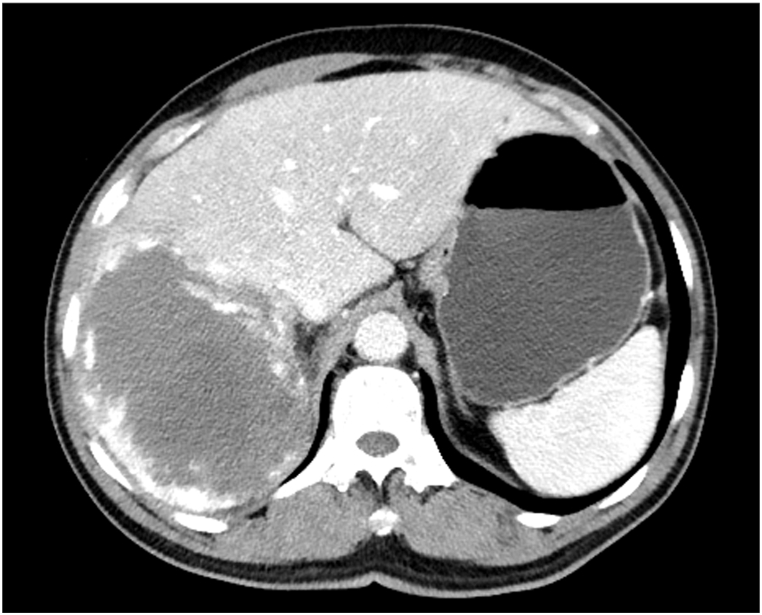
A 1.1-month follow-up CT demonstrated reduction in CHL size to 106.7 × 141.3 mm with residual enhancement in the periphery of the CHL.

**Fig. 4:**
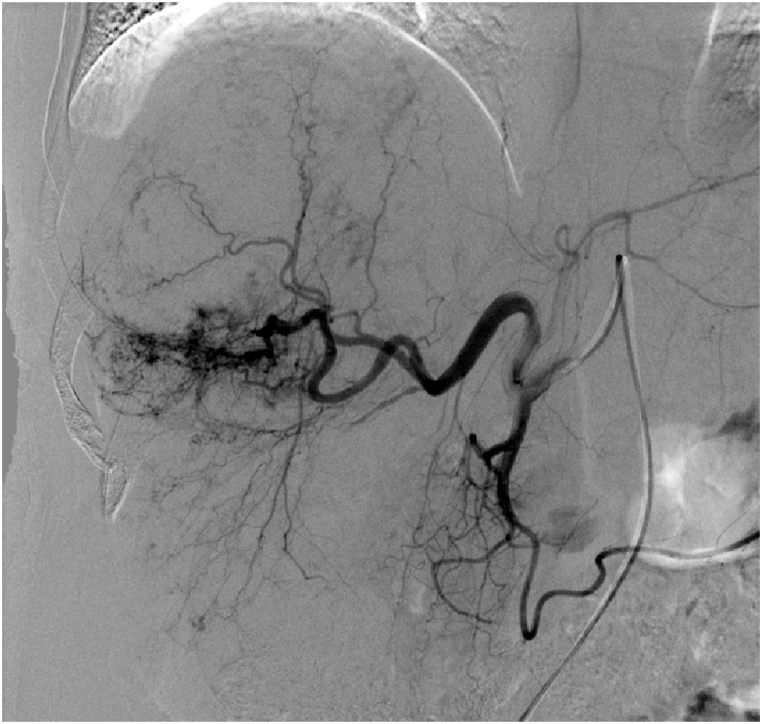
A second similar DEB-TACE was performed after 1.1 months.

**Fig. 5:**
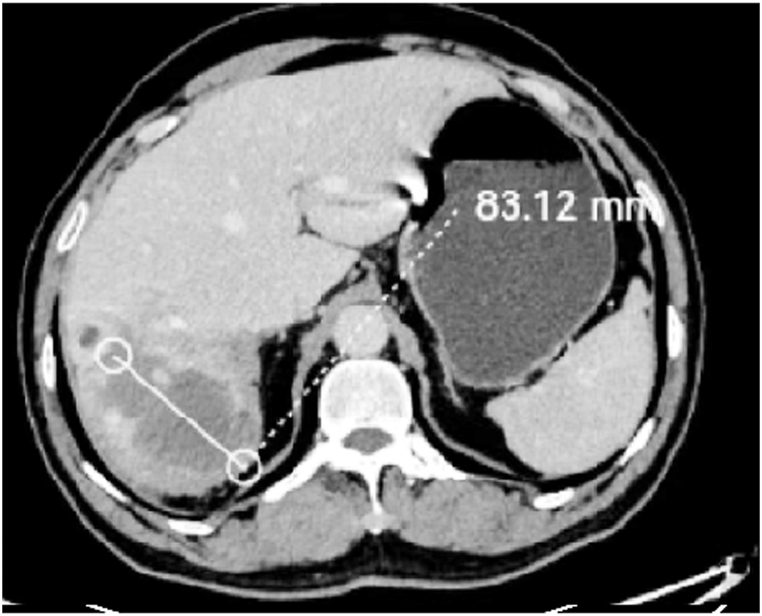
CT image obtained 4.6 months after the first DEB-TACE revealed considerable reduction in CHL size to 83.1 × 50.1 mm.

**Fig. 6:**
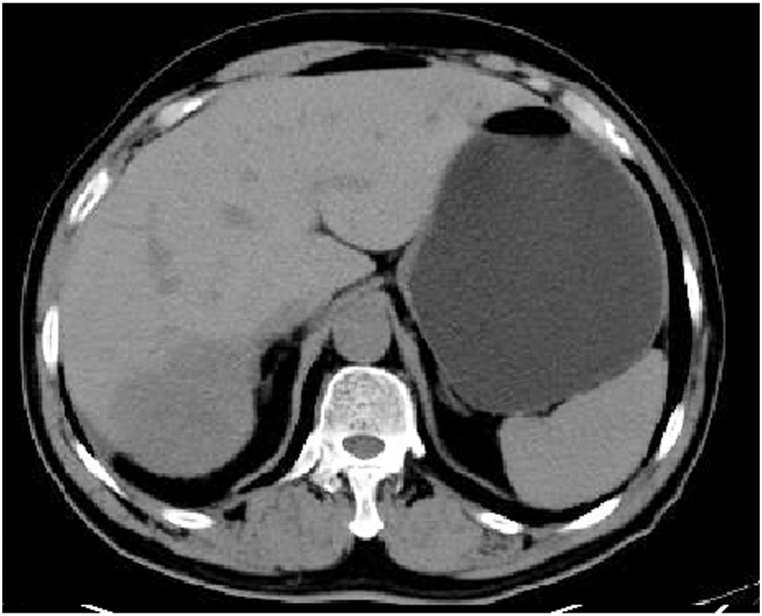
At 8.7 months of follow-up, the CHL size further reduced to 63.2 × 55.8 mm.

On the second day after embolisation, the patient experienced mild abdominal pain that resolved spontaneously within 3 days without pain medication. However, he still experienced cough and sputum symptoms, for which he felt anxious and returned for a follow-up review approximately 1.1 months after the embolisation. CT revealed a reduction in CHL size to 106.7 × 141.3 mm with residual enhancement in the periphery of the CHL (Fig. 3). Considering the residual enhancement of the lesion due to insufficiency of the previous embolisation as well as the patient's anxiety and strong demand for treatment, we performed a second similar DEB-TACE. One vial of 300–500-μm-diameter CalliSpheres microspheres loaded with 8-mg of pingyangmycin was used (Fig. 4), which resulted in further reduction in CHL size to 83.1 × 50.1 mm, as detected on the 4.6-month follow-up CT (Fig. 5). At the 8.7-month of follow-up, his clinical symptoms improved with no cough or sputum and the CHL size further reduced to 63.2 × 55.8 mm (Fig. 6).

## 3 Discussion

Histopathologically, CHL presents with several different sizes and thin-walled sinuses, resembling the shape of a cavernous sponge, and it also known as cavernous hsemangioma [2]. The intima of sinuses is composed of immature simple endothelium without any elastic or muscular layer. The hepatic artery is the most common feeding artery of the CHL. A pingyangmycin–lipiodol emulsion is injected into the sinuses through the feeding artery, which can be slowly cleared and stays in the sinuses for a considerable long time [2]. It has been clinically shown that pingyangmycin can destroy endothelial cells, which is also known as the devascular effect [6]. The pinyangomycin–lipiodol emulsion can also destroy the endothelial cells of the sinuses, resulting in the formation of numerous microthrombi in the sinuses, which eventually cause sinus fibrosis and atrophy of haemangioma [2]. This is the principle and mechanism of using the pingyangomycin–lipiodol emulsion for CHL treatment. Conversely, because a pingyangomycin–iodine oil emulsion can be rapidly washed out in normal liver tissue and blood vessels, it does not cause serious damage.

Transarterial embolisation has demonstrated acceptable efficacy with lower rates of post-procedural morbidity and mortality [2]. Pingyangomycin–iodine oil emulsion, polyvinyl alcohol, ethanol and iododol emulsions are commonly used embolic agents. However, polyvinyl alcohol may cause excruciating pain. Moreover, if the diameter of the solid particle embolisation agent is too large, it may embolise the small hepatic artery but fail to completely embolise the sinus and achieve the desired therapeutic effect. The ethanol and lipiodol emulsion alone can be deposited in the sinus, but it may be washed away by the blood flow, resulting in a poor therapeutic effect. Ethanol may cause reflux embolism and excruciating pain [7].

DEB-TACE has gradually been used as locoregional therapy for unresectable hepatocellular carcinoma. Although some controversy exists regarding the higher complete response rates of DEB-TACE and some authors recommend cTACE for this reason [5], our experience suggests that the unsatisfactory embolisation effect of DEB may be due to the inclusion of hepatocellular carcinoma with a diameter of <3 cm in their study, rather than the larger, unresectable hepatocellular carcinoma common in China. Furthermore, most researchers believe that DEB-TACE results in higher complete response, disease control rate and tumour necrosis rates than cTACE for unresectable hepatocellular carcinoma [3,4].

CalliSpheres beads with negative charges can form an absorptive interaction with positively charged drugs such as pirarubicin, raltitrexed and pingyangmycin [[8], [9], [10], [11]]. To our knowledge, this is the first case of a giant CHL that was treated using pingyangmycin-loaded DEB transarterial embolisation. In this case, DEB-TACE was performed as an alternative to reduce the size of the haemangioma along with a possible better necrosis of the CHL. Zeng et al. [2] reported a remarkable decrease in CHL diameter from 97 to 56 mm at 6 months after pingyangmycin–lipiodol embolisation. In our case, after two sessions of DEE embolisation treatments, the CHL size reduced significantly, with the maximum diameter decreasing from 187.5 mm before procedure to 63.2 mm at 8.7 months after DEB embolisation, suggesting a better necrosis rate than that reported by Zeng et al. [2]. As the transarterial embolisation was not sufficient with one vial of DEBs because of the large size of the CHL, we used two vials of 100- to 300-μm microspheres until the CHL staining disappeared. Additional embolisation is used to avoid possible washing away of DEBs or inadequate necrosis of the CHL due to insufficient embolisation.

Regarding safety, no procedure-related deaths or severe adverse events were observed during and after the procedure. In a previous study, incidence of complications of post-embolisation syndrome was significantly higher in the cTACE arm than in the DEB-TACE arm, whereas more biliary complications, such as biloma and biliary tract infection, were observed in the DEB-TACE arm [5].

This study has some limitations. First, deciding on a second session of DEB-TACE too early is not advisable, because more time is generally required to make this decision, considering that a significant reduction in CHL volume often occurs after 4–6 months of transarterial embolisation. Furthermore, although this preliminary report suggests that pingyangmycin-loaded DEB-TACE is feasible, further *in vitro* study is required to evaluate the efficient loading and binding of pingyangmycin with microspheres.

## 4 Conclusion

We report the first case of a giant CHL treated using DEB-TACE. Although DEB-TACE may be an effective and safe alternative for treating giant CHL, an *in vitro* study on the efficient loading and binding of pingyangmycin with microspheres and more comparative studies with larger samples are required to further confirm its safety and efficacy.

## Ethics statement

All procedures performed in studies involving human participants were in accordance with the ethical standards of the institutional and/or national research committee and with the 1964 Helsinki declaration and its later amendments or comparable ethical standards. This procedure was approved by the institutional review board.

## CRediT authorship contribution statement

**Yonghua Bi:** Writing – original draft, Resources, Project administration, Investigation, Conceptualization. **Jianzhuang Ren:** Writing – review & editing, Supervision, Project administration, Investigation, Formal analysis, Conceptualization. **Xinwei Han:** Writing – review & editing, Supervision, Project administration, Conceptualization.

## Declaration of competing interest

The authors declare that they have no known competing financial interests or personal relationships that could have appeared to influence the work reported in this paper.
